# Etching–Hydrophobicity Modification of Soybean Dreg Insoluble Dietary Fibers and Their Pickering Emulsion Stabilization Mechanisms

**DOI:** 10.3390/foods15152715

**Published:** 2026-08-01

**Authors:** Shuhan Ge, Haoyuan Li, Lingchao Wu, Wendan Jing, Hansong Yu

**Affiliations:** 1 College of Food Science and Engineering, Jilin Agricultural University, Changchun 130118, China; 18943915089@163.com (S.G.);; 2 Division of Soybean Processing, Soybean Research & Development Center, Chinese Agricultural Research System, Changchun 130118, China

**Keywords:** insoluble dietary fiber, etching modification, hydrophobic modification, Pickering emulsion, rheological behavior

## Abstract

Soybean dregs, one of the main byproducts of traditional soybean processing, are rich in insoluble dietary fiber (IDF), which possesses high mechanical strength and thermal stability. After surface reconstruction treatment, they have the potential to serve as a Pickering emulsion stabilizer, as these properties enable the particles to maintain their structural integrity at the oil–water interface. In this research, high-purity soybean dreg insoluble dietary fibers (HPSIDFs) were modified by alkaline H_2_O_2_, with oleic acid (OA) modified in a green synergistic modification. The surface of HPSIDF was etched to increase the reaction sites for subsequent hydrophobic modification, which enhanced the emulsification properties. Finally, the performance of the particles in Pickering emulsions and the emulsification mechanism were analyzed. The absolute value of the ζ-potential of the hydrophobically modified high-purity soybean dreg insoluble dietary fiber (O-HPSIDF) increased from 15.87 mV to 37.27 mV. The stability of the Pickering emulsion stabilized by O-HPSIDF was greatly enhanced; the droplet size of the emulsion decreased; the distribution was more uniform. The emulsifier particles formed a network structure at the interface, which imparted considerable mechanical strength to the emulsion, as evidenced by the increased storage modulus and viscosity.

## 1. Introduction

Soybean dregs, a major byproduct generated during traditional soybean product processing, are rich in dietary fiber, with contents reaching 84.47%. Notably, insoluble dietary fiber (IDF) constitutes 83.23% of this total. Soybean dreg insoluble dietary fiber (SIDF) mainly consists of cellulose, hemicellulose, and lignin. These fiber components cannot be digested by human digestive enzymes and are subsequently partially fermented in the large intestine or excreted directly from the body, and they have a wide range of applications in the food field [1]. It was shown that SIDF can hold up to 9.32 g/g of water, up to 6.41 g/g of oil, and has a solubility of up to 2.50 mL/g [2], and it exhibited good emulsification in the Pickering emulsion system [3,4,5]. SIDF can be used as green, natural, low-cost emulsifiers, thickeners, or food packaging materials, thereby reducing the food industry’s dependence on synthetic additives and providing a new pathway for the resourcing of agricultural by-products.

Pickering emulsion is an oil-in-water or water-in-oil emulsion that uses solid particles or colloidal particles that are insoluble in water and have certain hydrophilic and lipophilic properties as emulsifiers. In Pickering emulsions, solid particles irreversibly adsorb at the oil–water interface, forming a physical barrier that prevents droplet coalescence through steric hindrance. Unlike molecular emulsifiers, these particles stabilize emulsions through their amphiphilic properties and interfacial packing [6]. In addition, the emulsifiers of Pickering emulsions are mostly made of natural, biodegradable materials (e.g., cellulose, starch, etc.), which are less toxic [7] and more biocompatible than surfactants [7]. It also reduces production costs and minimizes waste, making it more environmentally friendly and economical. IDF is a good emulsifier for polysaccharide-based Pickering emulsions. Studies have shown that IDF from bamboo shoots can be used as emulsifiers to prepare stable Pickering emulsions [8], and IDF from enoki mushrooms can stabilize oil-in-water emulsions [9], indicating that insoluble dietary fibers have certain development potential in the field of emulsifiers for Pickering emulsions.

However, most insoluble dietary fibers have larger particles and a more compact structure [10], and these characteristics will weaken the emulsification of the particles, so they need to be modified in some way. The emulsifiability of IDF can be enhanced by modification treatments, such as reducing the droplet size [11], increasing the specific surface area, increasing the complexity of the surface spatial structure, regulating the type and number of chemical groups exposed on the surface, etc. At present, the modification of insoluble dietary fibers can be divided into four types according to the modification mechanism: physical modification, chemical modification, biological modification, and joint modification. The emulsifiability of grapefruit pomelo peel insoluble dietary fibers was significantly elevated after hydrophobic modification by octenyl succinic anhydride [12]. Citrus insoluble dietary fibers modified by ultrahigh pressure can form a more uniform interfacial adsorption network in Pickering emulsions and greatly improve the stability of the emulsion system [13]. For example, by modifying the SIDF at the molecular level, the balance between the hydrophilicity and hydrophobicity of the particles can be precisely regulated, and the emulsifying property of the particles can be further improved.

In this study, high-purity soybean dreg insoluble dietary fiber (HPSIDF) was prepared from SIDF and further processed by etching and hydrophobic modification. The alkaline H_2_O_2_ etching treatment performs two key functions: (1) It disrupts the compact fiber structure by cleaving ester and ether bonds between cellulose, hemicellulose, and lignin, creating pores and exposing internal hydroxyl groups; (2) it oxidizes some surface groups, generating additional reactive sites [14,15]. In the subsequent hydrophobic modification step, the carboxylic acid group of oleic acid (CH_3_(CH_2_)_7_CH=CH(CH_2_)_7_COOH) reacts with the exposed hydroxyl groups (-OH) of cellulose and hemicellulose through an esterification reaction, forming ester bonds (-COO-) and releasing water as a byproduct. Under acidic conditions (pH adjusted with HCl), the hydroxyl groups are protonated, facilitating nucleophilic attack on the carboxylic acid carbon, which promotes ester bond formation. This acid-catalyzed esterification mechanism has been well established for the modification of cellulose-based materials with fatty acids [16,17]. In this study, Pickering emulsions are stabilized with etch-hydrophobic synergistically modified HPSIDF (O-HPSIDF) as a solid emulsifier, and the role of O-HPSIDF in enhancing the stability of Pickering emulsions is analyzed, exploring the emulsification mechanism of particles in the emulsion system. This research innovatively employs a sequential alkaline H_2_O_2_ etching and oleic acid hydrophobic modification strategy to modify soybean dreg insoluble dietary fibers. Unlike previous single-modification approaches, the synergistic strategy combines surface roughening (to expose reactive sites) with hydrophobic grafting (to enhance amphiphilicity), offering a more effective and controllable means of tuning the interfacial properties of dietary fiber particles. This work not only highlights the potential for value-added utilization of soybean processing byproducts but also provides new perspectives for the design of plant-based Pickering stabilizers.

## 2. Materials and Methods

### 2.1. Materials and Reagents

The defatted soybean dregs used in the experiment (provided by Shandong Jiahua Health Products Co., Ltd, Liaocheng, China) contained approximately 70.3% dietary fiber (of which 90% was insoluble dietary fiber), 19.6% protein, 6.3% fat, and 3.8% ash on a dry weight basis. α-Amylase (4.0 × 10^4^ u/g, 0.25 g/mL), neutral protease (6.0 × 10^4^ u/g, 0.005 g/mL), and glucosidase (10.0 × 10^4^ u/g, 0.02 g/mL) were purchased from Beijing Solarbio Science Technology Co., Ltd. (Beijing, China). H_2_O_2_ solutions, OA, and other reagents were purchased from Tianjin Xinbote Chemical Co., Ltd. (Tianjin, China).

### 2.2. Preparation of HPSIDF, H-HPSIDF and O-HPSIDF

#### 2.2.1. Preparation of HPSIDF

HPSIDF was prepared using the method of Lyu et al. [18]. In brief, 15 g of defatted soybean dregs, 750 mL of distilled water, and 7.5 mL of α-amylase were mixed homogeneously and stirred at 95 °C for 35 min. The mixture was cooled to 60 °C. An additional 4.5 mL of neutral protease was added, and stirring was continued for 30 min at 60 °C. The mixture was then allowed to cool to room temperature, 150 mL of acetic acid solution (3 mol/L) was added, and the pH of the liquid in the conical flask was adjusted to 4.5 ± 0.02. Subsequently, 6 mL of glucoamylase was added, and the mixture was stirred at 60 °C for 30 min. Then, distilled water was added at 70 °C and stood for 2 h. To precipitate the dietary fiber, 95% food-grade ethanol (three times the total liquid volume) was added via thorough mixing. After standing for 45 min to allow complete precipitation, the precipitate was collected by filtration. The collected precipitate was air-dried at room temperature, ground into a fine powder, and sieved through a 200-mesh sieve to obtain HPSIDF with a purity greater than 90%.

#### 2.2.2. Preparation of H-HPSIDF

HPSIDF was etched using a H_2_O_2_ (3%) solution. Then, 2 g of HPSIDF and 40 mL of H_2_O_2_ (3%) solution were mixed well. The pH of the reactants was adjusted to 9.0 and then stirred at 55 °C for 4 h (600 r/min). After the pH of the reactants was adjusted to neutral, distilled water was added to 300 mL of the solution. The sediment was separated by centrifugation. After repeating the centrifugation step three times, the sediment was vacuum-lyophilized and ground, and finally, alkaline H_2_O_2_ etch-modified H-HPSIDF was obtained.

#### 2.2.3. Preparation of O-HPSIDF

O-HPSIDF was mixed with OA at a material–liquid ratio of 1:40, and 150 μL of 6 mol/L HCl solution was added. The reactants were dispersed by sonication at 800 W for 15 min and heated at 80 °C for 11 h. The precipitate was added to 40 mL of 95% ethanol and mixed well. The precipitate was centrifuged, and the step of adding ethanol and centrifugation was repeated three times. The precipitate was then washed by the addition of 40 mL of distilled water, followed by thorough mixing and centrifugation. This washing step was repeated twice. The final precipitate was collected, vacuum-lyophilized, and ground to a fine white powder to obtain O-HPSIDF.

#### 2.2.4. Preparation of the Pickering Emulsions

Referring to the method used by Liu et al. with slight modification [19], HPSIDF, H-HPSIDF, and O-HPSIDF were used as stabilizers to prepare Pickering emulsions, respectively. The amount of emulsifier added was 1.1%, and the ratio of the oil phase was 15%. Primary emulsification of the mixture was carried out using a high-speed shear dispersing emulsifier (10,000 r/min) before transferring it to an ultrasonic cell crusher and sonicating it for 14 min at 600 W. The prepared Pickering emulsions were stabilized by HPSIDF, H-HPSIDF, or O-HPSIDF particles.

### 2.3. Chemical Composition and Physicochemical Properties of the HPSIDF, H-HPSIDF and O-HPSIDF

#### 2.3.1. Water Holding Capacity (WHC)

WHC was determined according to the method reported previously [20]. HPSIDF, H-HPSIDF, and O-HPSIDF (0.5 g) were taken in a centrifuge tube, and 40 mL of distilled water was added. The centrifuge tubes were placed in a vertical position with the pointed bottom facing downwards for 24 h and centrifuged for 25 min (4500 r/min). The supernatant was poured off, and the mass of the remaining wet sediment was weighed and recorded. Repeat the above test 3 times and take the average value to calculate the water holding capacity according to the following formula:

$$\begin{array}{c} WHC\left(g/g\right)=\frac{W_{2}-W_{1}}{W_{1}} \end{array}$$

where W_1_ is the dry weight of the sample (g), and W_2_ is the weight of the wet sediment after centrifugation (g).

#### 2.3.2. Oil Holding Capacity (OHC)

OHC was determined according to a method reported previously [20]. HPSIDF, H-HPSIDF, and O-HPSIDF (0.5 g) were placed in a centrifuge tube, and 35 mL of edible oil was added. The centrifuge tubes were placed in a vertical stand for 24 h with the pointed bottom facing downwards and centrifuged for 25 min (4500 r/min). The supernatant was poured off, and the mass of the remaining wet sediment was weighed and recorded. Repeat the above test 3 times and take the average value to calculate the water holding capacity according to the following formula:

$$\begin{array}{c} OHC\left(g/g\right)=\frac{W_{2}-W_{1}}{W_{1}} \end{array}$$

where W_1_ is the dry weight of the sample (g), and W_2_ is the weight of the wet sediment after centrifugation (g).

#### 2.3.3. Water Swelling Capacity (WSC)

WSC was determined according to the method reported previously [20]. Then, 0.5 g of each of HPSIDF, H-HPSIDF, and O-HPSIDF was taken in a 50 mL graduated cylinder, and the initial volume was recorded.

Then, 30 mL of distilled water was added to the graduated cylinder, mixed well, and left to stand for 24 h. The volume of the sample after swelling at this point was recorded. The solubility was calculated according to the following formula:

$$\begin{array}{c} WSC\left(mL/g\right)=\frac{V_{2}-V_{1}}{W} \end{array}$$

where V_1_ is the initial volume of the sample (mL), V_2_ is the volume after swelling (mL), and W is the dry weight of the sample (g).

### 2.4. Particle Size, Droplet Sizes and ζ-Potential Measurements

Zetasizer Advance (Malvern , UK) was used to evaluate the particle size, droplet size, and ζ-potential measurements. The HPSIDF, H-HPSIDF, and O-HPSIDF samples (0.05 g) were dispersed in 10 mL of distilled water and vortex-mixed for 2 min to ensure complete dispersion. Then, 1 mL of the mixture was taken in the cuvette of the droplet size analyzer. Each sample was preheated in the machine at 25 °C for 2 min, and then, its droplet size value was measured. An aliquot of 1 mL of the dispersion was transferred to a disposable cuvette for droplet size measurement. Each sample was equilibrated at 25 °C for 2 min prior to measurement.

Particle size measurements were performed using a Malvern Zetasizer Advance (Malvern , UK) equipped with a 633 nm He-Ne laser. The instrument was configured with the standard 173° backscatter detection angle. For these measurements, the samples were dispersed in distilled water, and the particle size distribution was determined using the Mie theory-based volume distribution model. While the Zetasizer is primarily designed for submicron particles, it can measure particles in the micron range up to approximately 100 μm when configured with the appropriate optical setup and using the Mie scattering algorithm. To ensure reliable results, each sample was measured in triplicate, and the reported values represent the volume-weighted mean diameters (D[4,3]). The large particle size of HPSIDF (~82 μm) may also reflect the presence of aggregates in the dispersion, which were disrupted by the etching and modification treatments, contributing to the observed dramatic size reduction.

### 2.5. Fourier-Transform Infrared (FTIR) Analysis

Dried HPSIDF, H-HPSIDF, and O-HPSIDF were taken and mixed with KBr (1:100), which was dried to constant weight [21]. The mixture was ground for 10 min into a uniform fine powder using an agate mortar and pressed into uniform transparent discs of the size of a dime coin using a tablet press. The samples were detected by Fourier transform infrared spectroscopy (Cary-660, Linli Instruments Co., Ltd., Shanghai, China).

### 2.6. X-Ray Diffraction (XRD)

X-ray diffraction was performed using PANalytical X’pert3 (Malvern Panalytical instruments Ltd., UK). The crystal structures of HPSIDF, H-HPSIDF, and O-HPSIDF were observed under measurement conditions of 5–90° and a scanning speed of 2°/2 min [22]. The crystallinity of HPSIDF and H-HPSIDF was calculated using the following equation:

$$\begin{array}{c} Dc\left(\%\right)=\frac{I_{002}-I_{am}}{I_{002}}\times 100 \end{array}$$

where the variables are defined as follows: Dc—degree of crystallinity; I_002_—diffraction intensity in the crystalline region (2θ = 20.78°); I_am_—diffraction intensity in the amorphous region (2θ = 18.00°)

### 2.7. Scanning Electron Microscopy (SEM)

The microscopic morphology of HPSIDF, H-HPSIDF, and O-HPSIDF was observed using a S4800 (Hitachi, Tokyo, Japan ). The dried HPSIDF and H-HPSIDF were gently fixed on conductive adhesive tape, lightly blown off the unadhered powder on the surface, and placed in an ion sputtering apparatus for gold spraying. Then, the sample stage was transferred to a scanning electron microscope, the surface microforms of the samples were observed under an accelerating voltage of 10 kV, and the images were taken at 500, 1 k, 2 k, 5 k, and 20 k magnifications.

### 2.8. Thermogravimetry–Differential Scanning Calorimetry (TG-DSC)

Thermogravimetric analysis (TGA) and differential scanning calorimetry (DSC) were performed simultaneously using a Q600 simultaneous thermal analyzer (TA Instruments, New Castle, DE, USA). The samples (3 mg) were heated from 10 °C to 600 °C at a rate of 10 °C/min under a nitrogen atmosphere (flow rate: 50 mL/min). The TGA curves recorded mass loss as a function of temperature, while the DSC curves recorded heat flow changes, allowing the identification of thermal transitions such as glass transition, crystallization, and decomposition events.

### 2.9. Pickering Emulsion Stability Test

Pickering emulsions were prepared as described in Section 2.2.4 and subjected to various stability tests: (1) storage at room temperature for up to 15 days; (2) centrifugation at 6000, 8000, and 10,000 r/min for 15 min; (3) freeze–thaw cycling at −80 °C for 12 h followed by thawing at room temperature; (4) heat treatment in a water bath at 65 °C and 85 °C for 25 min; and (5) pH adjustment to 3, 4, 5, 6, 7, 8, and 9, followed by storage for 15 days. After each treatment, the emulsion stability was evaluated by visual observation of creaming, phase separation, and sedimentation.

### 2.10. ζ-Potential Measurements of Pickering Emulsions

ζ-potential of the emulsion samples was measured using a Zetasizer Advance (Malvern Panalytical, UK). The same volume of HPSIDF-, H-HPSIDF-, and O-HPSIDF-stabilized emulsions was taken and diluted with distilled water at a 1:10 ratio (*v*/*v*); 1 mL was taken in a potentiometric cuvette, and the potential values were measured.

### 2.11. Microstructure of Pickering Emulsion

#### 2.11.1. Inverted Fluorescence Microscope

The emulsions were detected by an inverted fluorescence microscope according to a method reported previously [12]. Droplet size, aggregation, and flocculation of the emulsions were observed using Leica DMILLED (Leica, Wetzlar, Germany). The emulsions prepared from HPSIDF, H-HPSIDF, and O-HPSIDF were taken separately and diluted at a ratio of 1:10. The diluted solution was dropped on a slide, and the droplet size, aggregation, and flocculation of the emulsions were observed using an inverted fluorescence microscope (Leica DMILLED, Leica Microsystems, Wetzlar, Germany).

#### 2.11.2. Laser Confocal Microscope

The microstructural characteristics of the emulsions, including droplet size, aggregation, and flocculation, were observed using an inverted fluorescence microscope (Leica DMILLED, Leica Microsystems, Germany) [3]. The microstructural characteristics of the emulsions were further examined using a laser confocal microscope (Leica Stellaris 5, Leica Microsystems, Germany). The Pickering emulsions were stained using three dyes (Nile Red, Calcofluor white, and Nile Blue), and microstructural photographs of the emulsions were obtained at a scale of 50 μm.

### 2.12. Rheological Measurements

Rheological measurements were performed using a DHR-2 rheometer (TA Instruments, USA) equipped with a 40 mm diameter parallel plate geometry with a 1.0 mm gap. All measurements were conducted at 25 °C. The emulsion samples prepared using HPSIDF, H-HPSIDF, and O-HPSIDF were placed on a rheometer operating table with a 1.0 mm gap, and the shear rate was 0.01–100 s^−1^ at 25 °C. The variation in the apparent viscosity of emulsions with respect to shear rates was determined and plotted as static rheological curves.

Dynamic rheological properties were analyzed by applying oscillatory frequencies ranging from 0.1 to 100 rad/s at a fixed oscillatory strain of 1%, which was confirmed to be within the linear viscoelastic region from preliminary strain sweep tests. The value of the oscillatory strain was fixed at 1%, and the oscillation frequency range was 0.1–100 rad/s. The variation of the oscillation frequency of the emulsions concerning the energy storage modulus, G′, and loss modulus, G″, was determined and plotted as a dynamic rheological curve.

### 2.13. Statistical Analysis

Data were analyzed using IBM SPSS Statistics 23 software, and images were drawn using Origin 2021. Three parallel experiments were set up for each experiment, and the experimental data were presented in the form of mean ± standard deviation (*n* = 3, independently repeated experiments). The significance of differences between groups was determined by one-way analysis of variance (ANOVA) combined with Tukey’s post hoc test, and the significance level was set at α = 0.05. According to the statistical labeling convention, when the comparison between groups showed *p* < 0.05, the difference was identified using a different lowercase letter (e.g., a, b, and c); if the two groups shared at least one common letter (e.g., group ‘ab’ versus group ‘a’), this indicated that the difference between the comparison groups was not statistically significant.

## 3. Results and Discussion

### 3.1. The Microstructural Properties of the Prepared O-HPSIDF

**Figure 1:**
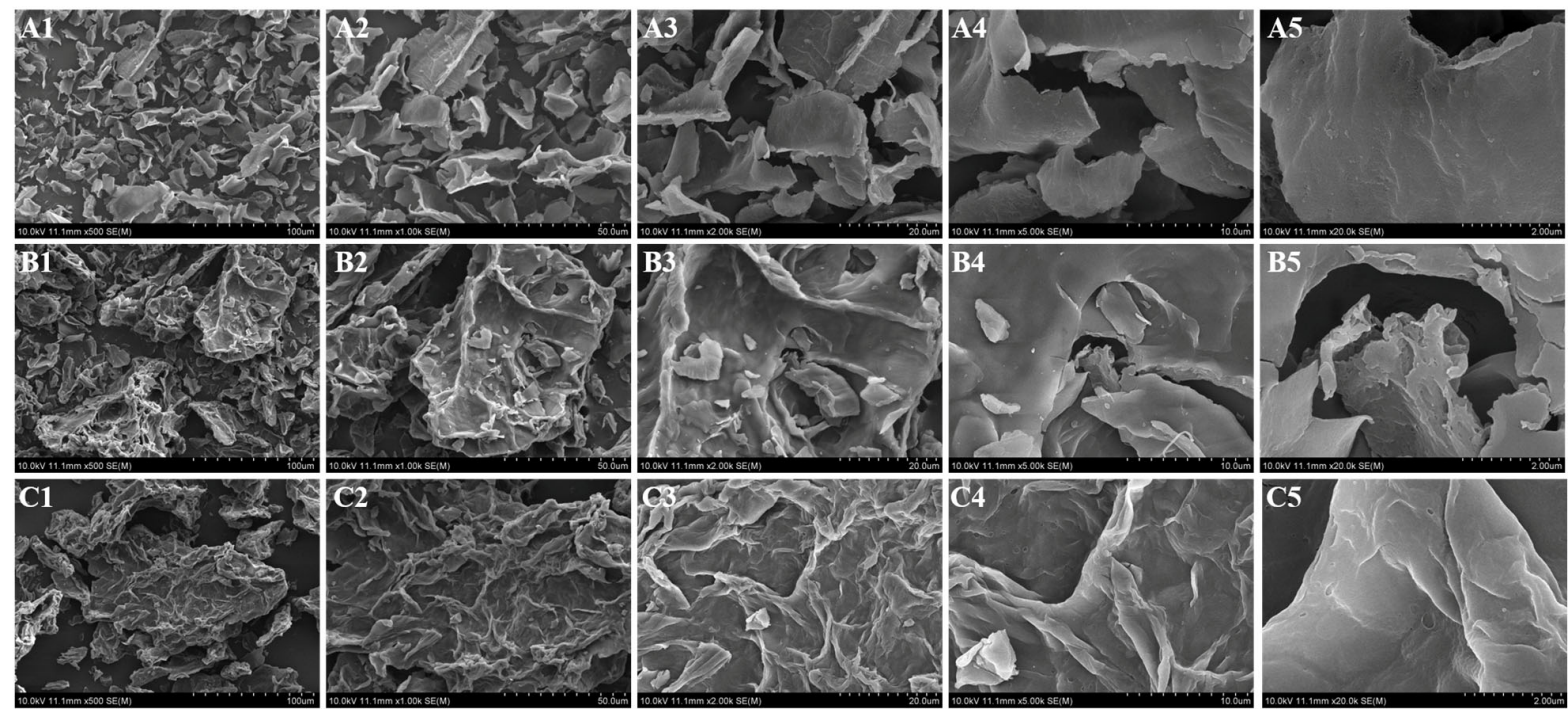
(**A1**–**A5**) SEM image of HPSIDF. (**B1**–**B5**) SEM image of H-HPSIDF. (**C1**–**C5**) SEM image of O-HPSIDF (magnification: 500× (**A1**,**B1**,**C1**); 1000× (**A2**,**B2**,**C2**); 2000× (**A3**,**B3**,**C3**); 5000× (**A4**,**B4**,**C4**); 20,000× (**A5**,**B5**,**C5**)).

The surface micro-morphology of HPSIDF, H-HPSIDF, and O-HPSIDF is shown in Figure 1. The untreated HPSIDF is in the form of a curled sheet with a relatively smooth surface and a dense structure. The surface of H-HPSIDF after H_2_O_2_ etching treatment showed a large number of holes and grooves, which changed the original smooth surface into a spongy structure with a complex spatial structure, and the internal structure was exposed through the holes and gaps. The hydrophobically treated O-HPSIDF still has a more complex spatial structure than the HPSIDF, but it had shallower grooves and fewer holes than H-HPSIDF.

The reason for the formation of the complex structure of H-HPSIDF may be that alkaline H_2_O_2_ disrupted the structure of H-HPSIDF to some extent. This caused the formation of more pores and richer folds on the particle surface, exposing more internal structures and thus providing more modification sites for the next hydrophobic modification. The structural changes of O-HPSIDF may be due to the newly formed modified layer during hydrophobic modification of oleic acid, clogging up the original hole. In addition, the treatment process leads to a certain degree of collapse of the spatial structure of the particles. However, O-HPSIDF still showed a complex morphology with multiple folds; the complexity of the surface space structure was higher than that of HPSIDF, and such a surface structure could enhance the emulsification of O-HPSIDF.

**Figure 2:**
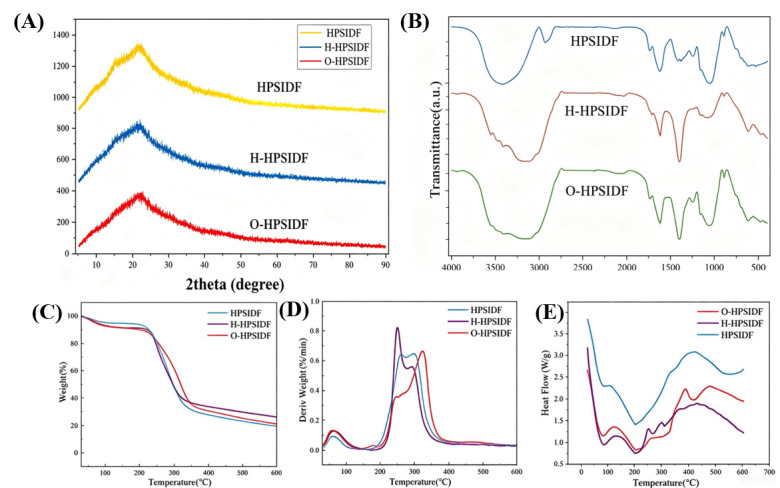
(**A**) X-ray diffraction of HPSIDF, H-HPSIDF, and O-HPSIDF. (**B**) FT-IR spectra of HPSIDF, H-HPSIDF, and O-HPSIDF. (**C**–**E**) TG-DSC image of HPSIDF, H-HPSIDF, and O-HPSIDF. (**C**) Thermogravimetric curve (TG). (**D**) First-order differential thermogravimetric curve (DTG). (**E**) Heat flow versus temperature curve (DSC).

As shown in Figure 2A, the XRD patterns of HPSIDF, H-HPSIDF, and O-HPSIDF show typical cellulose type I characteristics. This indicates that the main components of HPSIDF, H-HPSIDF, and O-HPSIDF are natural cellulose type I structures. The 2θ angles of the two are relatively close to each other, indicating that they have similar crystalline types, and the modification treatment will not change the crystalline types of HPSIDF. The crystallinity index of HPSIDF was calculated to be 29.11%, while H-HPSIDF and O-HPSIDF showed values of 29.00% and 29.055%, respectively. The slight decrease in crystallinity after etching (from 29.11% to 29.00%) suggests that the treatments primarily affected the amorphous regions of the cellulose structure, while the crystalline regions remained largely intact. The crystallinity of O-HPSIDF (29.05%) remained essentially unchanged compared to H-HPSIDF, indicating that the subsequent hydrophobic modification with oleic acid did not further disrupt the crystalline structure. This is consistent with previous reports on alkaline hydrogen peroxide treatment of dietary fibers, which showed that the treatment preferentially disrupts amorphous cellulose and hemicellulose without significantly altering the crystalline cellulose I structure.

A comparison of the Fourier transform infrared spectral characteristics of HPSIDF, H-HPSIDF, and O-HPSIDF samples is shown in Figure 2B. The broad absorption band in the range of 3200–3600 cm^−1^ is a typical feature of hydroxyl (-OH) stretching vibrations, which originates from the hydrogen-bonding network of primary (C6-OH) and secondary (C2/C3-OH) hydroxyl groups in the cellulose molecular chain, as well as from hydroxyl groups in hemicellulose and lignin. This assignment is well-established in the literature on plant-based dietary fibers [23,24]. The double peaks located near 2920 cm^−1^ and 2850 cm^−1^ correspond to the antisymmetric and symmetric telescopic vibrational modes of methyl (-CH_3_) and methylene (-CH_2_-) [25], which may originate from smidgeon residual protein fractions in the fibers of the soybean dregs. The characteristic absorption peaks in the 1650–1750 cm^−1^ region correspond to carbonyl (C=O) stretching vibration modes, suggesting the presence of carbonyl structures in the sample and that such groups may originate from lignin fractions [24,26,27]. The absorption peak near 1740 cm^−1^ may originate from the vibration of the ester group, and the absorption peak in the range of 1450–1600 cm^−1^ is closely related to the backbone vibration of the lignin benzene ring (C=C aryl ring stretching), suggesting that the lignin component is preserved [28]. The results suggest that oleic acid was successfully incorporated onto the fiber surface, enhancing the amphiphilic balance of the particles. Further studies using XPS or elemental analysis would be beneficial to definitively confirm covalent grafting.

As shown in Figure 2C–E, the thermal properties of the materials can be evaluated by thermogravimetric analysis, and the thermogravimetric curves (TGs) and first-order differential thermogravimetric curves (DTGs) are shown in Figure 2. The weight-loss peaks near 60 °C correspond to the evaporation of surface-adsorbed water. The major decomposition occurring between 250 °C and 370 °C is attributed to the thermal decomposition (pyrolysis) of cellulose, hemicellulose, and lignin components. The appearance of two overlapping peaks in the DTG curves suggests the sequential decomposition of hemicellulose (which decomposes at lower temperatures, 250–290 °C) and cellulose (which decomposes at higher temperatures, 300–370 °C) [14,29]. After etching treatment, the maximum decomposition rate of H-HPSIDF increased significantly, which may be related to the disruption of chemical bonds, making the originally tightly bound components more susceptible to thermal degradation. Notably, the maximum weight-loss peak temperature of O-HPSIDF shifted to a higher temperature (from 326 °C to 341 °C), and the maximum decomposition rate decreased, indicating that the oleic acid modification layer enhanced the thermal stability of the fibers. This is consistent with previous reports on hydrophobically modified polysaccharides.

### 3.2. The Processing Characteristics of the Prepared O-HPSIDF

**Figure 3:**
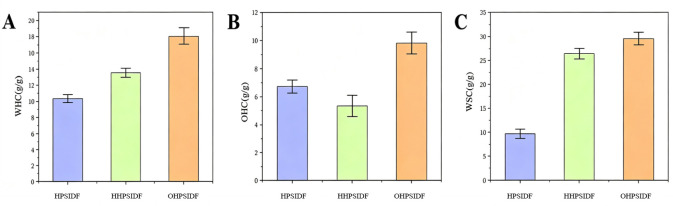
(**A**) WHC of HPSIDF, H-HPSIDF, and O-HPSIDF. (**B**) OHC of HPSIDF, H-HPSIDF, and O-HPSIDF. (**C**) WSC of HPSIDF, H-HPSIDF, and O-HPSIDF.

HPSIDF, H-HPSIDF, and O-HPSIDF were tested and analyzed for WHC, OHC, and WSC, as in Figure 3A–C. The results showed that after being treated by the etching of H_2_O_2_, the WHC and WSC of H-HPSIDF increased, and the OHC slightly decreased. After being modified by hydrophobization, the OHC of O-HPSIDF was significantly elevated. The enhanced OHC of O-HPSIDF is consistent with findings by Gao et al. [12], who reported that OSA modification of pomelo peel IDF significantly increased oil binding capacity due to the introduction of hydrophobic groups. Similarly, Yang et al. [13] observed that ultra-high pressure-modified citrus IDF exhibited improved oil holding capacity, which they attributed to structural unfolding and exposure of hydrophobic residues. The increase in WHC and WSC of H-HPSIDF could be attributed to the fact that the etching of the HPSIDF surface by H_2_O_2_ produced more pores on the surface and exposed more hydrophilic groups, such as active hydroxyls, and the sites at which water could bind were increased. The decrease in OHC may be due to the destruction of lipophilic groups on the surface and inside of HPSIDF and a decrease in oil binding sites. The increase in OHC of OHPSIDF may be due to the modification of the surface of O-HPSIDF by oleic acid, which resulted in an increase in lipophilic groups and consequently an increase in OHC.

As shown in Table 1, the droplet size of soya bean residue insoluble dietary fiber (HPSIDF) before modification was 82.43 ± 0.27 μm, the droplet size of H-HPSIDF after the etching treatment with H_2_O_2_ was reduced to 7.04 ± 0.18 μm, and that of O-HPSIDF was reduced to 4.99 ± 0.22 μm. This result may be attributed to the alkaline environment of H_2_O_2_, which destroys the covalent bonds between cellulose and hemicellulose, and lignin was partially solubilized and oxidized by the alkaline H_2_O_2_ [14,15], providing more hydrophobic reaction sites for the next step of oleic acid modification. The oleic acid molecules affected the state of dietary fiber molecules during the modification of O-HPSIDF, which disrupted the large-scale particle state and aggregation morphology of the particles, resulting in certain rupture of the particles and leading to a reduction in droplet size.

The ζ-potential of HPSIDF was −15.87 ± 0.32 mV, that of H-HPSIDF was −17.21 ± 0.45 mV, and that of O-HPSIDF was −37.27 mV ± 1.52 mV, which indicated that the surface charge density of the fiber particles increased after modification. The increase in the absolute value of the ζ-potential of H-HPSIDF may be due to the fact that the etching treatment destroys the surface of the particles, resulting in the exposure of more negatively charged groups, such as hydroxyl and carboxyl groups. The increase in the absolute value of the ζ-potential of O-HPSIDF may be due to the oleic acid esterifying the surface of the O-HPSIDF, which introduces new charged groups and increases the charge density on the surface of the O-HPSIDF, increasing the absolute value of the ζ-potential. The absolute value increases. The higher absolute value of ζ-potential can inhibit the agglomeration between particles, improve the stability of the liquid system, and enhance the emulsification of O-HPSIDF in Pickering emulsions.

### 3.3. Evaluation of the Interfacial Activity and Stability of the Prepared O-HPSIDF

**Figure 4:**
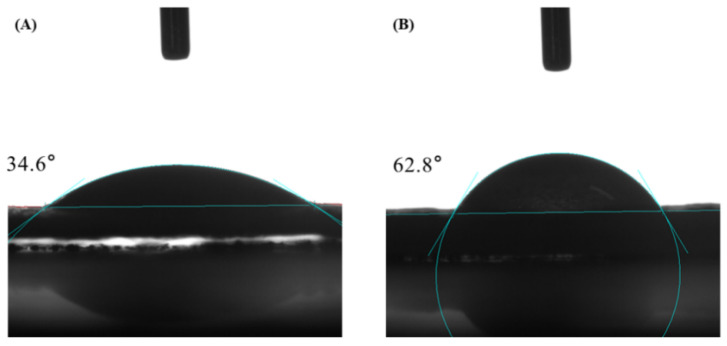
Comparison of contact angle between HPSIDF and HHPSIDF ((**A**): contact angle of HPSIDF; (**B**): contact angle of HHPSIDF).

The three-phase contact angles of HPIDF, HHPIDF, and OHPIDF were measured. As shown in Figure 4, the water contact angles of both HPSIDF and HHPSIDF are less than 90°. The droplets on the tablets in the images are water, and the tangent angle at the contact point between the droplet and the tablet surface is less than 90°, indicating that the material is hydrophilic, whereas an angle greater than 90° indicates hydrophobicity; the smaller the contact angle, the better the hydrophilicity of the material. According to the figure, both HPSIDF and HHPSIDF exhibit good hydrophilicity, with the contact angle of HPSIDF being 34.6° and that of HHPSIDF being 62.8°. The closer the contact angle is to 90°, the better the emulsifying properties. The three-phase contact angle test results indicate that after etching treatment, the emulsifying properties of HHPSIDF are also improved. The contact angle was used as an evaluation index to optimize the preparation process of OHPIDF. Under the preparation conditions of a heating temperature of 80 °C, a heating time of 11 h, an HCl addition of 150 μL/g, an ultrasonic power of 500 W, and an ultrasonic time of 15 min, the contact angle of OHPIDF approached 70.0°, indicating a significant increase in its hydrophobicity.

**Figure 5:**
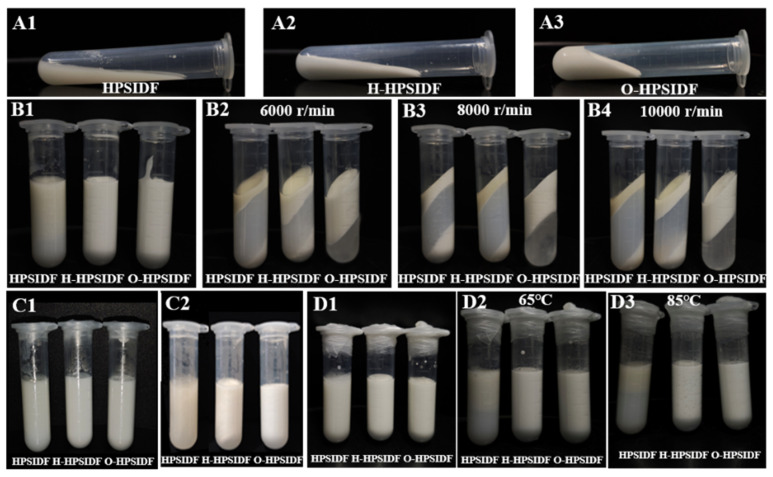
(**A1**–**A3**) Comparison of the fluidity of HPSIDF, H-HPSIDF, and O-HPSIDF emulsions. (**B1**–**B4**) Changes in centrifugal treatment of HPSIDF, H-HPSIDF, and O-HPSIDF emulsions. (**C1**,**C2**) Changes in HPSIDF, H-HPSIDF, and O-HPSIDF emulsion freeze–thaw treatments. (**C1**) Freeze–thaw before treatment. (**C2**) Freeze–thaw after treatment. (**D1**–**D3**) Changes in heat treatment of HPSIDF, H-HPSIDF, and O-HPSIDF emulsions.

To determine the interfacial activity of the prepared O-HPSIDF, it was dispersed in two water–oil phases to form a Pickering emulsion. As shown in Figure 5A1–A3, different flow properties can be observed for different Pickering emulsions expressed in containers with the same tilt angle. The thinnest emulsion in the figure is the Pickering emulsion of HPSIDF, followed by the Pickering emulsion of H-HPSIDF. The most viscous emulsion state is the Pickering emulsion of O-HPSIDF, which indicates that the O-HPSIDF emulsion has a strong resistance to external deformation, probably due to the increased affinity of O-HPSIDF with the oil phase after hydrophobic modification, resulting in stronger interactions at the oil–water interface and the formation of a more robust network to better stabilize the emulsion system.

The Pickering emulsion, stabilized by O-HPSIDF, was subjected to centrifugation, freeze–thaw, and heat treatment to determine its stability. Centrifugal stability simulates the ability of emulsions to withstand extreme external forces in everyday life due to transportation, violent shaking, etc. It also accelerates the evaluation of stability for very long storage times. As shown in Figure 4B1–B4, the three emulsions showed varying degrees of stratification after centrifugation. The HPSIDF emulsion showed the most severe stratification, with a large amount of turbid aqueous phase precipitating in the middle layer. The H-HPSIDF emulsion retained more of the emulsified layer, with some turbid aqueous phase also observed in the middle layer, and the O-HPSIDF emulsion retained most of the emulsified layer. This phenomenon may be attributed to the hydrophobic modification of O-HPSIDF, which greatly enhanced the binding ability of O-HPSIDF to biphases, forming a strong spatial structure to resist the strong irritating external force caused by centrifugation.

Freeze–thaw treatment allows us to examine the adsorption effect of emulsifier particles and the stability of the emulsions when the ambient temperature difference is large. As shown in Figure 5C1 and C2, HPSIDF emulsions showed large oil droplets, turbidity, and delamination after freeze–thaw, and the emulsions underwent demulsification. H-HPSIDF emulsions showed significant delamination after freeze–thaw, and the emulsions became cloudy. O-HPSIDF showed only slight turbidity at the bottom after freeze–thaw, and there was no obvious delamination. The freeze–thaw stability of O-HPSIDF emulsions is superior to that reported for unmodified okara IDF emulsions [30], which showed significant phase separation after freeze–thaw cycling. This improvement is likely due to the enhanced interfacial affinity and network-forming ability of O-HPSIDF, which prevents ice-crystal-induced droplet coalescence.

As shown in Figure 5D1–D3, when the three emulsions were heated at 65 °C, the HPSIDF emulsion showed obvious delamination, and the H-HPSIDF emulsion and O-HPSIDF emulsion did not change significantly. When the three emulsions were heated at 85 °C, a large amount of delamination appeared in the HPSIDF emulsion, the H-HPSIDF emulsion became inhomogeneous, and the O-HPSIDF emulsion still maintained a uniform milky white color. This indicates that the hydrophobically modified O-HPSIDF particles can still stabilize the Pickering emulsion system at higher temperatures and show excellent emulsification ability, and the Pickering emulsions prepared by this emulsifier have good thermal stability, which confirms that the hydrophobic modification can enhance its emulsification properties.

**Figure 6:**
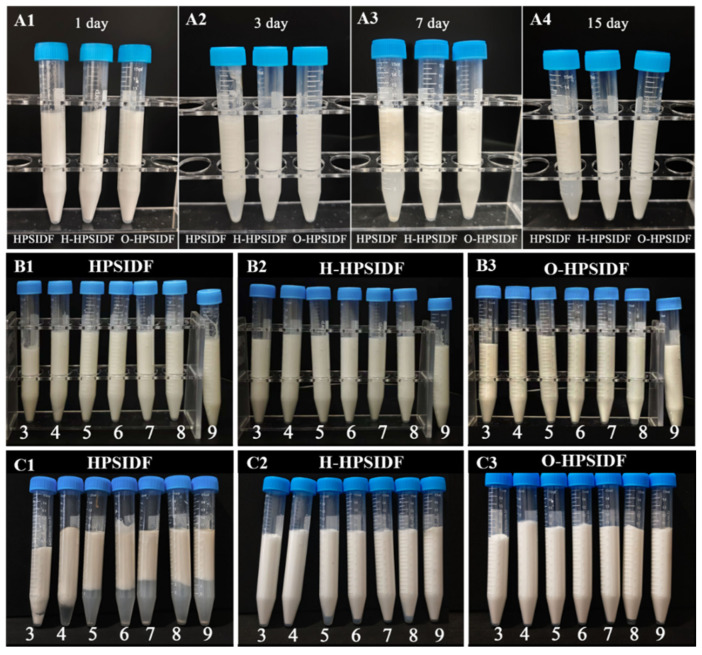
(**A1**–**A4**) Changes in HPSIDF, H-HPSIDF, and O-HPSIDF emulsions after storage. (**B1**–**B3**,**C1**–**C3**) Changes in HPSIDF, H-HPSIDF, and O-HPSIDF emulsions after storage at different pH values. (**B1**–**B3**) Day 1. (**C1**–**C3**) Day 15.

The storage stability of the Pickering emulsion to some extent reflects the emulsification performance of the emulsifier; therefore, the storage stability of the emulsion was tested over a period of 15 days. As shown in Figure 6A1–A4, the condition of the Pickering emulsions of HPSIDF, H-HPSIDF, and O-HPSIDF remained good on the first day, with all three emulsions being homogeneous white emulsions with no visible precipitation or layering. On the third day, the bottom of the HPSIDF emulsion showed some delamination, and a turbid water phase precipitated below, while the H-HPSIDF and O-HPSIDF emulsions did not show delamination. On the seventh day, the turbid water phase precipitated from the emulsion of HPSIDF increased further, and the emulsions of H-HPSIDF and O-HPSIDF still did not show delamination. On the 15th day, the HPSIDF emulsion maintained the original layering phenomenon, the H-HPSIDF emulsion was turbid but not layered, and the O-HPSIDF emulsion was still a uniform milky white color and did not produce the phenomenon of layering or turbidity, indicating that the emulsifying property of O-HPSIDF was enhanced after modification. The modified O-HPSIDF may have enhanced the role of grasping and attaching the two phases at the interface, which greatly improved the stability of this Pickering emulsion.

The pH values have a certain degree of influence on the storage stability of the Pickering emulsions. As shown in Figure 6B1–B3 and C1–C3, HPSIDF, H-HPSIDF, and O-HPSIDF were not in a state of precipitation and delamination at different pH values on the day of emulsion preparation. With the increase in storage time up to 15 days, delamination of HPSIDF gradually occurred, and the severity of delamination increased with the increase in pH, indicating that Pickering emulsions prepared with HPSIDF were less stable under alkaline conditions. The Pickering emulsion prepared by H-HPSIDF showed less delamination only at the bottom under a higher alkaline environment. The Pickering emulsions prepared by O-HPSIDF showed no delamination and precipitation at all pH values, indicating that the Pickering emulsions stabilized by O-HPSIDF possessed strong tolerance to environmental pH.

### 3.4. Microstructural Characterization and Physicochemical Properties of Pickering Emulsions Stabilized by O-HPSIDF

**Figure 7:**
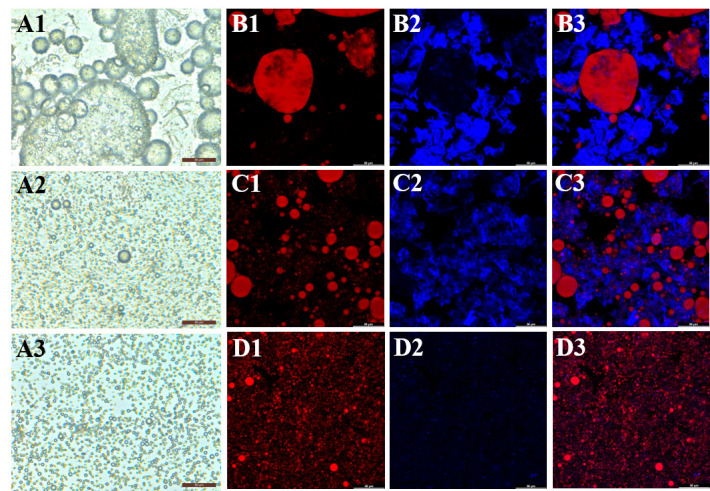
(**A1**–**A3**) Inverted fluorescence microscope image of HPSIDF emulsion, H-HPSIDF emulsion, and O-HPSIDF emulsion at 40× magnification. (**A1**) HPSIDF emulsion. (**A2**) H-HPSIDF Pickering emulsion. (**A3**) O-HPSIDF emulsion. (**B1**–**D3**) Laser confocal microscope images of HPSIDF emulsion, H-HPSIDF emulsion, and O-HPSIDF emulsion (scale bar: 50 μm). (**B1**–**B3**) HPSIDF emulsion. (**C1**–**C3**) H-HPSIDF emulsion. (**D1**–**D3**) O-HPSIDF emulsion.

In the HPSIDF emulsion, the size of the droplets was not uniform. There were large oil droplets with extremely large sizes; some smaller droplets were aggregated near the large droplets; some of the large droplets tended to be aggregated; there were fibrous HPSIDFs with large sizes observed in the emulsion (Figure 7A1–A3). The H-HPSIDF emulsion’s droplet size is relatively more uniform, and the size of the droplets is greatly reduced; there are only a few large droplets that exist between droplets. The spacing between droplets is relatively more uniform, and the distribution state is more regular. The droplet size of the Pickering emulsion prepared by O-HPSIDF is the most uniform; there are almost no obvious larger droplets, the distribution of droplets is dense and uniform, and the spacing between droplets is more consistent, indicating that O-HPSIDF can best stabilize the Pickering emulsion.

The microstructure of the emulsion was observed by laser confocal microscopy, in which the blue part was the emulsifier, and the red part was the oil droplets, as shown in Figure 6. The Pickering emulsion prepared by HPSIDF contained oil droplets of a large size, and the size was very uneven. The distribution of the emulsifier is also not uniform. The combination of emulsifier and oil droplets is relatively loose; both have the phenomenon of similar aggregation phenomenon, and the overall emulsion structure is rough. The size of the oil droplets of the emulsions prepared by H-HPSIDF was smaller and more homogeneous than that of the HPSIDF emulsions, and the emulsifier was more uniformly distributed around the oil droplets, which may be due to the smaller droplet size, more surface-active groups, and more complex spatial structure of the H-HPSIDF, which enhanced its original emulsifying properties. Both emulsifier and oil droplets in the O-HPSIDF emulsion showed highly uniform dispersion; the oil droplets had the smallest size and the most uniform distribution, the fibers showed highly homogeneous distributions, and the emulsion structure was fine. This may be because the hydrophobic-treated O-HPSIDF retains a part of the emulsifying property of H-HPSIDF, and the subsequent hydrophobic modification further enhances its emulsifying ability so that it can better adsorb at the oil–water interface, reduce the interfacial energy, and form a dense and stable interfacial membrane, which can effectively hinder the oil droplets from approaching each other to achieve a highly stable emulsification effect.

**Figure 8:**
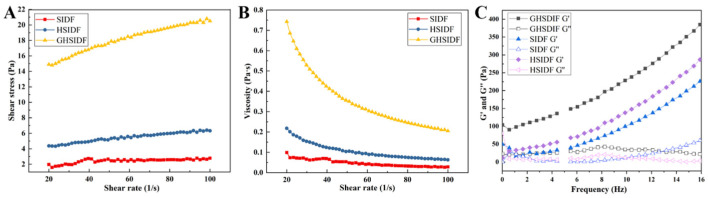
Rheological analysis graphs for HPSIDF emulsions, H-HPSIDF emulsions, and O-HPSIDF emulsions. (**A**) Plot of shear stress versus shear rate. (**B**) Plot of viscosity versus shear rate. (**C**) Plot of storage modulus and loss modulus versus frequency.

Figure 8 shows the rheological measurement images of the emulsions prepared using HPSIDF, H-HPSIDF, and O-HPSIDF as emulsifiers, respectively. The flow curves (Figure 8A) exhibited downward curvature with a decreasing slope, indicating shear-thinning (pseudoplastic) behavior, which is typical of non-Newtonian fluids [31,32]. The viscosity curves (Figure 8B) confirmed this behavior, showing a decrease in viscosity with increasing shear rate. The O-HPSIDF emulsion exhibited the highest initial viscosity and the largest absolute decrease in viscosity with increasing shear rates, indicating the presence of a highly structured network that is readily disrupted under shear. The H-HPSIDF emulsion has medium initial viscosity and a medium decrease in viscosity with shear rate. The HPSIDF emulsion exhibited the lowest initial viscosity and a relatively small absolute decrease in viscosity with increasing shear rate, suggesting that the emulsion had a weaker network structure compared to the modified samples. The reason for the pseudoplastic fluid is that the fluid contains intertwined long chains or macromolecules, which can form a certain structure to hinder the flow of the object in the stationary state [33,34]. The shear-thinning behavior observed in all emulsions is characteristic of colloidal suspensions containing aggregated particles. Under low shear rates, the network structure formed by particle–particle interactions and the interfacial film imparts high viscosity to the system. As the shear rate increases, the applied shear forces overcome the interparticle interactions, breaking down the aggregated particle network and aligning the particles along the flow direction, resulting in a decrease in apparent viscosity [10,35]. This disruption of the network structure, rather than particle alignment, is the primary mechanism responsible for the shear-thinning behavior in Pickering emulsions stabilized by insoluble dietary fiber particles. Similar observations have been reported for other Pickering emulsion systems stabilized by polysaccharide-based particles.

As seen in Figure 8C, the energy storage modulus of the three emulsions is greater than the loss modulus, indicating that the three emulsions are elastic-based emulsions with a certain solid-like characteristic and better stability. When the frequency increases, the energy storage modulus of all three emulsions rises significantly, indicating that the ability of the emulsions to resist deformation at high frequencies is enhanced, and the elastic properties are more prominent. The O-HPSIDF emulsion had the largest energy storage modulus, and its stabilized emulsion has the strongest elasticity, optimal structural stability, and high deformation resistance. H-HPSIDF emulsion occupies second place, indicating that etching can change the interfacial properties of particles to some extent. The results show that the hydrophobic modification further optimizes the adsorption and arrangement of the particles at the interface, strengthens the firmness of the adsorption of O-HPSIDF at the interface, and forms a high-stability Pickering emulsion, which is capable of counteracting a certain amount of external forces, verifying the positive effect of hydrophobic modifications on the improvement of the rheological properties and stability of emulsions.

In summary, the Pickering emulsion stabilized by O-HPSIDF has high consistency and high structural strength, and its internal structure is not easily destroyed when subjected to external forces, which can better maintain the stability of the system. This may be because the particles of untreated HPSIDF are more compact and larger, and the number of hydrophilic and lipophilic groups exposed on the surface is lower, which stabilizes the emulsion droplets less. The surface of the etch-treated H-HPSIDF became loosely porous and reduced in size, exposing more hydrophilic groups and improving its interaction with emulsion droplets, which led to an increase in its ability to stabilize the emulsion. O-HPSIDF was hydrophobized to introduce new hydrophobic groups on the surface, making it more amphiphilic. The smaller droplet size and dense interfacial coverage observed in confocal microscopy (Figure 7) suggest that O-HPSIDF particles effectively adsorb at the oil–water interface. The higher storage modulus and viscosity of the O-HPSIDF emulsion indicate the formation of a stronger particle network in the continuous phase, thus preventing the escape of droplets [36]. This structure also enhances the interaction between the emulsion droplets, which improves the viscosity and deformation resistance of the emulsion [37], resulting in better stability and structural strength of the emulsion. However, it is acknowledged that these conclusions are indirectly inferred; direct measurements such as interfacial tension, interfacial rheology, or quantitative confocal image analysis would provide more definitive evidence of the proposed stabilization mechanism in future studies.

## 4. Conclusions

In conclusion, etching–hydrophobicity synergistic modifications can optimize the emulsifying capacity of insoluble dietary fiber particles. Etching increases surface roughness and reactive sites, thus providing conditions for hydrophobic modification. The hydrophobic modification further optimizes the emulsifying capacity of the fibers by introducing new hydrophobic groups, which results in a Pickering emulsion with high stability. The O-HPSIDF-stabilized Pickering emulsions with the smallest and uniformly distributed droplet sizes had the highest stability of the emulsions. The analysis shows that hydrophobic treatment can enhance the adsorption ability of the particles at the interface of the two phases, and O-HPSIDF can form a layer of compact interfacial film, which further forms a three-dimensional network structure. This plays a good role in immobilizing the oil and water phases, and at the same time, it can effectively buffer the impact of external forces so that the emulsion’s stability is greatly improved. The etch-hydrophobic modified O-HPSIDF can be used as a natural, green, and safe solid emulsifier to replace chemical synthetic emulsifiers for future applications in the field of plant-based and clean-label food products, and its good deformation resistance can make it a unique stabilization advantage in long-distance transportation. This study deepens the understanding of the surface modification mechanism of dietary fibers and provides ideas for the development of new, efficient, and controllable surface modification processes for dietary fibers and the design of Pickering emulsifiers.

## Figures and Tables

**Table 1:** Particle size and zeta potential of HPSIDF, H-HPSIDF, and O-HPSIDF.

Sample	Droplet Sizes (μm)	ζ-Potential (mV)
HPSIDF	82.43 ± 0.27 a	−15.87 ± 0.32 a
H-HPSIDF	7.04 ± 0.18 b	−17.21 ± 0.45 b
O-HPSIDF	4.99 ± 0.22 c	−37.27 ± 1.52 c

Note: Values = mean ± standard deviation (*n* = 3). Within the same column, different lowercase letters indicate a significant difference (*p* < 0.05), while the same letter indicates a non-significant difference (*p* > 0.05).

## Data Availability

The original contributions presented in this study are included in this article. Further inquiries can be directed to the corresponding authors.

## Abbreviations

HPSIDF	High-purity soybean dreg insoluble dietary fibers
H-HPSIDF	Alkaline H_2_O_2_ etch-modified high-purity soybean dreg insoluble dietary fiber
O-HPSIDF	Oleic acid modified high-purity soybean dreg insoluble dietary fiber
